# Supratherapeutic International Normalized Ratio causing Nephropathy: A Rare Adverse Effect of Warfarin

**DOI:** 10.7759/cureus.5201

**Published:** 2019-07-22

**Authors:** Muhammad Shabbir Rawala, Amna Saleem Ahmed, Muhammad Y Khan, Muhammad Nauman Riaz, Amr Eltoukhy

**Affiliations:** 1 Internal Medicine, Charleston Area Medical Center, Charleston, USA; 2 Internal Medicine, Jinnah Medical & Dental College, Karachi, PAK; 3 Internal Medicine, Unitypoint Health, Rock Island, USA; 4 Internal Medicine, Rapides Regional Medical Center, Alexandria, USA

**Keywords:** warfarin, nephropathy, supratherapeutic inr

## Abstract

There are many common causes of nephropathy (abnormal pathology of kidneys) such as diabetes, hypertension, autoimmune, and drugs. Amongst the drugs, warfarin has recently been recognized to cause nephropathy in rare cases. Warfarin-related nephropathy (WRN) is clinically defined as an increase in serum creatinine of 0.3 mg/dl within one week of international normalized ratio (INR) being greater than 3.

A 61-year-old male was referred by his primary care physician (PCP) for having complaints of elevated creatinine associated with hematuria for one month. On evaluation with computed tomography (CT) of the abdomen/pelvis, it was revealed that he had small non-obstructing stones. The creatinine had increased from a baseline of 2.03 mg/dl to 6.8 mg/dl. Hemoglobin (Hb) had decreased from a baseline of 12.8 gm/dl to 8.1 gm/dl, the INR was 3.52. On subsequent days, the patient's renal function did not improve with fluids and supportive measures. Workup was unremarkable; therefore, a kidney biopsy was done. The biopsy specimen concluded the diagnosis of WRN. The patient was started on prednisone without any effect and then intermittent hemodialysis.

Our case highlights the rare instance in which the cause of nephropathy is warfarin. If an early diagnosis had been made, the patient might have had a better prognosis; therefore, it is essential to have a high index of clinical suspicion when a patient presents with supratherapeutic INR and acute kidney injury (AKI) not getting better.

## Introduction

Warfarin is one of the widely used anticoagulants in different disorders to prevent thrombosis in clinical settings [1-2]. It has been the most prescribed anticoagulant in the United States which currently has more than 30 million prescriptions filled each year [3-4]. It works by competitively inhibiting vitamin K epoxide reductase enzyme complex and hence disrupting the extrinsic clotting cascade [3,5]. Warfarin requires close monitoring due to its narrow therapeutic range and dreaded complication of hemorrhage [3]. Reported adverse effects of warfarin related to the kidney include acute interstitial nephritis, hemorrhage, leukocytoclastic vasculitis, spontaneous cholesterol embolization in patients with diffuse atherosclerosis, and ischemic acute tubular necrosis secondary to massive blood loss [2,6].

A rather new complication described by Brodsky et al. is warfarin-related nephropathy (WRN), clinically diagnosed as an episode of unexplained acute renal injury, characterized by a 0.3 mg/dl increase in serum creatinine within one week of international normalized ratio (INR)> 3.0 in patients on warfarin therapy, without evidence of hemorrhage [3-5,7]. Initially, WRN was hypothesized to be secondary to tubular obstruction by RBC casts resulting in the back flux of glomerular filtrate and increased renal cortical interstitial fluid pressure compressing neighboring tubules [2]. However, studies have also suggested hemoglobin (Hb) nephrotoxicity in its pathogenesis [2].

The primary mechanism of the clinical picture of acute kidney injury (AKI) in WRN is believed to be due to glomerular hemorrhage along with a widespread renal tubular obstruction by red blood cell casts, without significant hemodynamic changes [2,4-6]. With the growing use of warfarin in a wide array of clinical settings like chronic heart failure and chronic atrial fibrillation, WRN is not as rare an entity, occurring in 16% of non-chronic kidney disease (CKD) patients and 37% of CKD patients on warfarin, with at least a single reported episode of raised INR> 3.0 [8-9].

We hereby report a case of a 61-year-old Caucasian male with complaints of elevated creatinine associated with hematuria for one month on warfarin therapy.

## Case presentation

A 61-year-old Caucasian male was referred by his primary care physician (PCP) for having complaints of elevated creatinine associated with hematuria for one month. His other co-morbid conditions were coronary artery disease, atrial fibrillation, hypertension, CKD, iron deficiency anemia, osteoarthritis, mitral valve replacement, and newly diagnosed benign prostate hypertrophy.

He had been to another hospital twice before presenting to the emergency department (ED) where he had been evaluated with cystoscopy that had ruled out any bladder pathology. On evaluation with computed tomography (CT) of the abdomen/pelvis, it was revealed that he had small non-obstructing stones and was told to follow up as an outpatient. He did follow up with his PCP who saw that his creatinine had increased from a baseline of 2.03 mg/dl to 6.8 mg/dl. On initial evaluation at our institution's ED, his creatinine was 6.8 mg/dl, Hb had decreased from a baseline of 12.8 gm/dl to 8.1 gm/dl, the INR was 3.52. CT abdomen/pelvis demonstrated no evidence of acute pathology, negative for obstructive uropathy, crossed unfused renal ectopia, and left kidney is in the abdomen.

**Figure 1:**
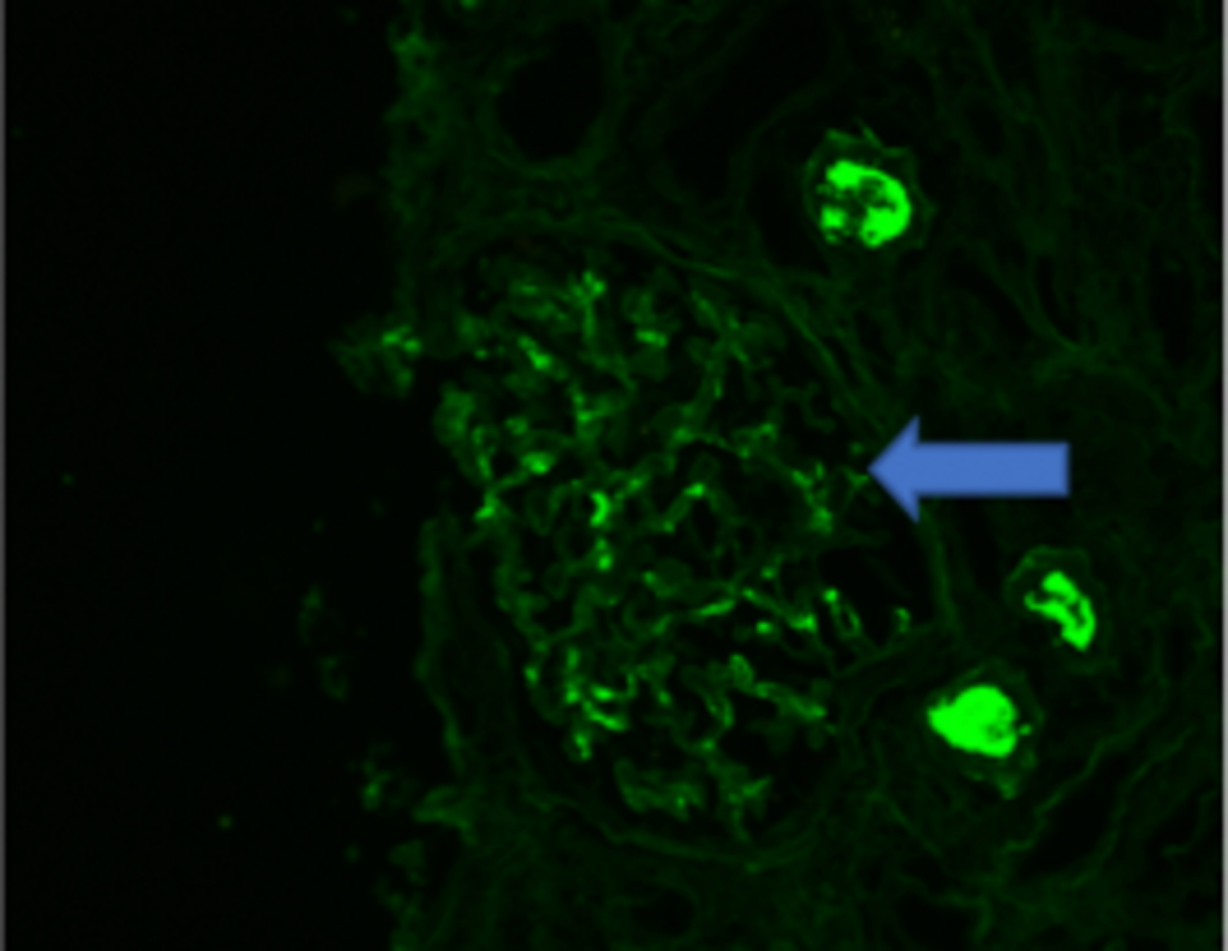
Immunoglobulin A (IgA) deposition on the nephron (arrow)

**Figure 2:**
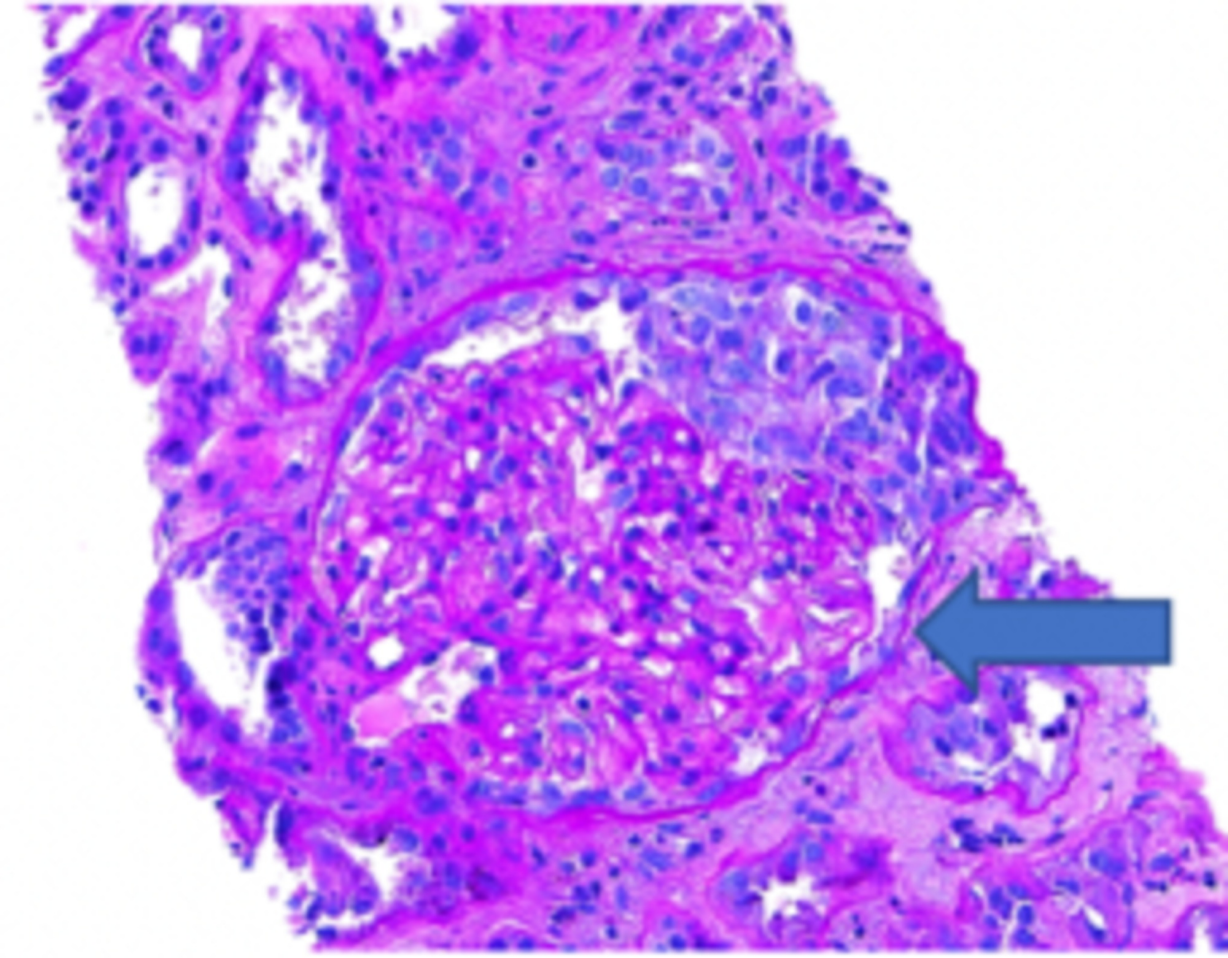
Specimen showing focal crescentic glomerulonephritis (arrow)

**Figure 3:**
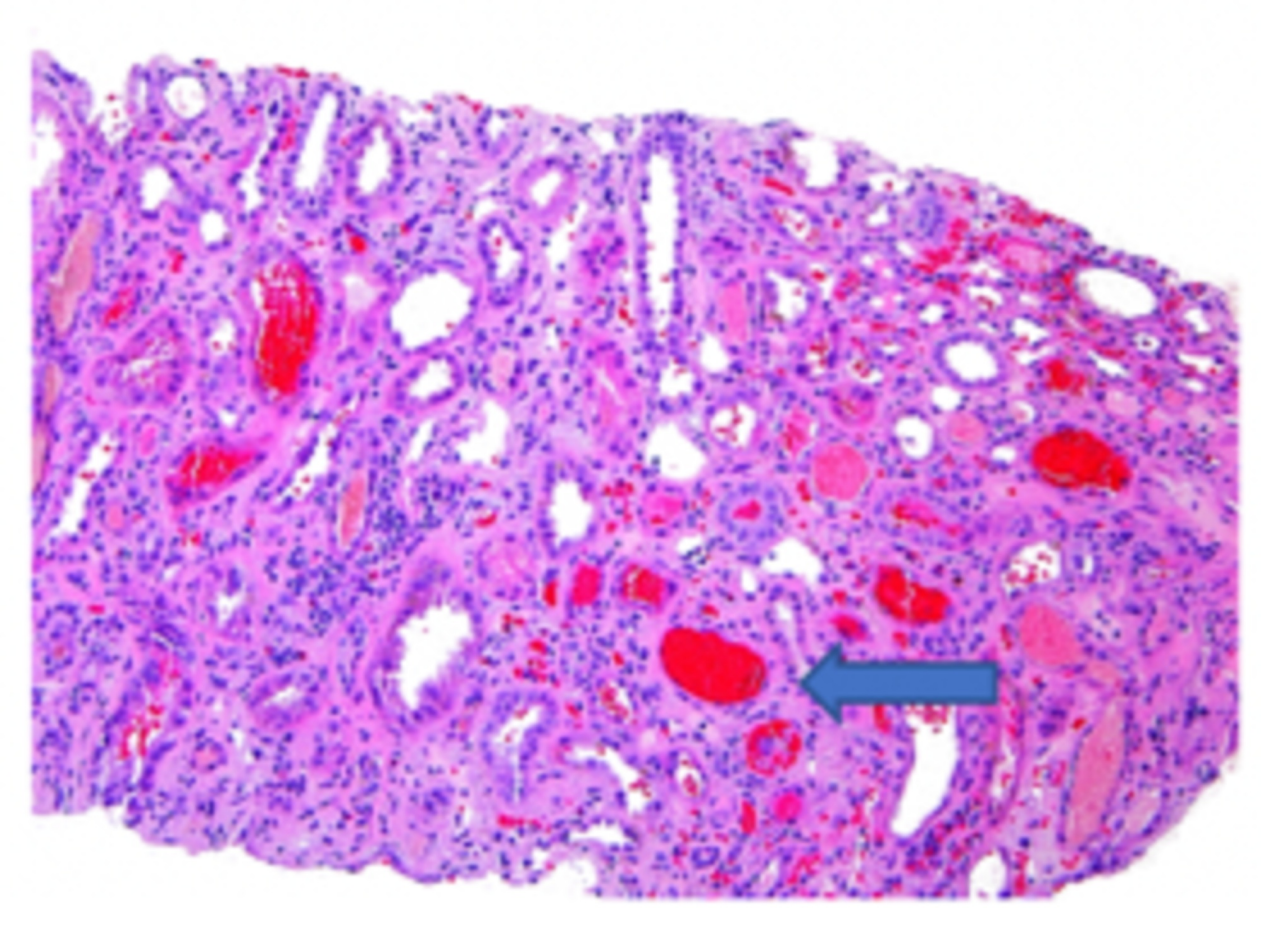
Red blood cell cast formation (arrow) in the nephron

Based on laboratory/imaging data, it was thought that the hematuria and AKI were secondary to kidney stones that the patient had passed. On subsequent days, the patient's renal function did not improve with fluids and supportive measures. Workup for vasculitis, infection, and protein electrophoresis were negative; it was decided to proceed with a renal biopsy. The biopsy specimen revealed minimal immunoglobulin A (IgA) deposition, as seen in Figure 1, and focal crescentic glomerulonephritis, as seen in Figure 2, with diffuse tubular injury and red blood cells (RBC) cast formation ultimately concluding the diagnosis of WRN (Figure 3).

## Discussion

Due to warfarin's narrow therapeutic index and differences in dose requirement based on genetics, diet, and environmental factors, ascertainment for WRN occurrence in each race remains debatable [5]. However, risk factors reported include age, diabetes mellitus, hypertension, low serum basal albumin level, coexisting congestive heart failure (CHF), and high serum aspartate transaminase (AST) at post INR elevation, the mechanism for which are unclear but are believed to associated with higher INR after initiating warfarin [3-5]. As 97% of warfarin is bound to albumin in the body, hypoalbuminemia and CHF (results in dilution hypoalbuminemia) lead to accumulation of unbound warfarin which therein results in over-anticoagulation.

The mechanism that promotes AKI in warfarin therapy includes atheroembolism, interstitial nephritis, and direct effects of warfarin on the glomerulus [4]. Brodsky et al. also showed that WRN appears early during the warfarin course and does not necessarily require severe warfarin coagulopathy, signified by the lack of difference in INR levels of WRN patients compared to non-WRN [2,4]. In addition, there was no association established between increases in INR with creatinine elevation in patients [4]. A study published in 2011 makes the association of concomitant use of warfarin with certain antimicrobial agents like sulfonamide derivatives mimicking WRN, while concomitance with aspirin increases the risk of WRN, perhaps the case in our patient [4,10].

The association of microscopic hematuria as a common side effect of warfarin treatment in the absence of acute kidney disease has been reported in 20% of the patients [6,11]. While Santos et al. report hematuria in warfarin-treated patients as an indication of underlying kidney disease [2]. Upon review of the literature, it suggests that warfarin therapy along with structurally abnormal glomerular basement membrane (GBM), manifested as thick or thin GBM, cause severe glomerular hematuria due to obstruction by RBCs, eventually leading to acute renal failure which could be superimposed by WRN [5,12-13]. Kabir et al. also observe that the gross hematuria seen in patients undergoing warfarin anticoagulation as an actual indication of an underlying glomerulopathy like a structurally abnormal GBM or an inflammatory glomerulopathy [12].

Heber et al. concluded with their study that even the mildest glomerular disease combined with warfarin-induced coagulopathy may lead to hematuria along with the accumulation of RBCs within tubules [6]. Moreover, diminished urinary flow further exacerbates the condition by forming occlusive casts out of intratubular RBCs leading to AKI, concluding the event to be a serious complication of warfarin therapy [6].

Brodsky et al. demonstrated an accelerated progression of CKD in patients with an increase in creatinine with INR >3.0. WRN is confirmed to be associated with a significant increase in mortality rate hence underrepresenting the population affected [3-4]. However, Janek writes about the selection bias in WRN studies highlighting the selection of subjects with INR greater than 3.0 essentially produces a cohort enriched subjects with acute illnesses which eventually could be responsible for the increased creatinine [14].

Moreover, it is also apparent that this increased mortality risk is also related to multiple comorbidities and risk factors for AKI present in these patients, which could eventually lead to acute deterioration and mortality [4,14]. As Nephrologists typically refrain from ordering a kidney biopsy in warfarin-treated patients due to the risk of hemorrhage, the disease could have progressed to an advanced stage by the time of diagnosis [4].

Brodsky et al. in 2011, developed a rat model of ablative nephropathy to aid understanding of the pathogenesis of WRN [7]. The study reported that severe anticoagulation of these animal models reproduced findings such as elevated creatinine levels, hematuria, occlusive RBC cats, and acute tubular injury, similar to those in patients of WRN [7]. The model also helped suggest alternate theories in the mechanism of WRN. One such theory exhibits warfarin interfering with activation of a product of growth specific gene 6, which in turn affects glomerular mesangial cells and glomerular hemodynamics, further exacerbating any underlying kidney pathology. Researchers also observed increased apoptosis of glomerular endothelial cells [7].

In 2012, the same animal was used to study warfarin treatment, which produced a dose-dependent rise in creatinine correlating with an increase in prothrombin time [9]. The model also confirmed that more advanced stages of ablation were more susceptible to WRN [9]. This study discovered that vitamin K prevented the creatinine increase and its associated renal changes while also completely reversing the coagulopathy by returning creatinine to baseline, perhaps highlighting the importance of coagulopathy in the pathogenesis of WRN. Other findings of the research include observation of RBC and focal RBC casts in animal models within normal ranges of INR (between 2 and 3) suggesting a possibility of WRN occurrence in humans even in therapeutic INR levels of warfarin [9]. Furthermore, authors also observed that a single episode of WRN did not affect CKD progression in animal models, but this could be credited to the fact that only young animals were selected in the study in contrast to the typical scenario in the human population which mostly involves elderly patients with multiple comorbidities [9].

While multicenter studies are awaited to understand the prevalence and other characteristics of susceptible patients like estimated glomerular filtration rate, urinary protein and albumin excretion, quantitative analysis of urinary red blood cell, protein S and C, and others [3]. Healthcare personals must be aware of the serious complication of warfarin therapy and carefully monitor coagulation parameters, and kidney function simultaneously; while any evidence of gross hematuria should immediately prompt discontinuation or at least reduced dosage of anticoagulants [2].

## Conclusions

We present a rare adverse effect of warfarin. Physicians should be judicious and prompt in recognizing nephropathy caused by supratherapeutic INR as timely discontinuation may lead to favorable outcomes. It also highlights that physicians should closely monitor patients as nephropathy may lead to permanent renal damage or worsening of existing CKD.
